# The Cult of the Operating Theatre

**Published:** 1903-06-20

**Authors:** 


### The Cult of the Operating Theatre.

Very large sums of money are spent upon the operating theatre in public institutions as well as in many nursing homes. Yet the genesis of the operating theatre is quite modern, and its exclusive use to the purpose after which it is named is only a matter of a few years. The distressing scenes which accompanied every operation in the days before the invention of chloroform rendered it necessary for a room to be set apart for the use of the surgeon. Our ancestors were neither nervous nor squeamish, and in the older hospitals the operating room is often to be found in the centre of the building, and with wards upon either side. In many cases it was merely a room set aside for an occasional purpose, but when students or onlookers attended a detached building would often be provided. Thus we read in Heister's "Medieal and Chirurgical Observations" that Dr. Rau operated in 1707 at the Gasthaus, in Amsterdam, in a room appointed for the purpose, and that "as he was tall, fat and corpulent he used to pull off his coat to prevent his being too warm, but kept on his waistcoat, tying about him an apron in which was a pocket containing the instruments." Operations were not the sole use to which the room was put, because in many institutions meetings of various kinds were held in the operating theatre, and there is a survival of this custom at St. Bartholomew's Hospital, where surgical consultations are still held once a week in the presence of a large body of students and on patients upon whom it is not proposed to operate, at any rate immediately. Sometimes the theatre was put to a less legitimate purpose in other large institutions, and when operations were not being performed upon the living body a course of operative surgery would be carried out on the dead subject.

To-day matters are very different, and rightly so. The advance of Listerian principles has taught every surgeon that his theatre should be reserved exclusively for purposes of operation, and many surgeons hold that each operator should have his own operating theatre, just as he has his own wards and his own nursing staff. It is probable that as time goes on such a scheme will become general at any rate in the more enlightened and wealthy of our hospitals. But it is somewhat of a question whether the very splendour of modern operating theatres does not give a sense of false security, unless care is taken to carry out all those minute precautions by which alone an operation can be conducted to a successful issue. The best results are being constantly obtained in the oldest theatres, which are apparently least adapted to their present requirements; whilst the very first cases in a theatre upon which untold money has been spent have been known to end most disastrously, for the patient has died of septic infection after a so-called minor operation.

In the presence of such a calamity the noble maxim adopted by Gœthe should be followed. It runs:—

"Nichts taugt Ungeduld
Noch weniger Reue:
Jene vermehrt die Schuld,
Diese schafft neue."

Impatience is of no service, still less remorse. The one increases the offence, the other makes new ones, for the surgeon becomes anxious and unstrung. A resolute attempt should be made to discover the cause of such a disastrous failure when all seems so fair on the outside. The more obvious sources of contamination should be first eliminated, the more hidden may then be looked for. The cleaning of such a theatre has been known in one place to be entrusted to a charwoman fresh from the lavatories or the floor of the post-mortem room; whilst in another custom has allowed the lotions used at the operation to be prepared by the unwashen hands of the hall porter; in yet another infection has been carried from the dirty coat of the bearer carrying the patient back to the ward, when a carelessly or badly applied dressing has slipped in the process of transference from the operating table to the stretcher. It is clear, therefore, that the personal factor of the surgeon is not necessarily predominant at an operation, but that the environment is also of importance and that he and his staff must be Argus-eyed to detect and remedy every flaw in the arrangements.

